# Wildfire‐Specific PM_2.5_ Asthma Risk Across a Disproportionately Burdened Pediatric Population in Northern California

**DOI:** 10.1029/2025GH001530

**Published:** 2026-02-11

**Authors:** Rebecca Sugrue, Stephanie Holm, Andrew Nguyen, Morgan Ye, Rosana Aguilera, Dayna Long, Tarik Benmarhnia, Rosemarie De La Rosa, Neeta Thakur

**Affiliations:** ^1^ Department of Medicine University of California San Francisco San Francisco CA USA; ^2^ Stephanie Holm Consulting Vancouver BC Canada; ^3^ Scripps Institution of Oceanography University of California San Diego San Diego CA USA; ^4^ Department of Pediatrics University of California San Francisco San Francisco CA USA; ^5^ School of Public Health University of California Berkeley Berkeley CA USA

**Keywords:** air pollution, asthma, epidemiology, wildfire smoke

## Abstract

Wildfire smoke events have increased in severity and impact over the past two decades and are expected to remain a major contributor to air pollution especially in the Western U.S. Children and communities with pre‐existing environmental and socioeconomic disadvantages, such as those in Alameda and Contra Costa Counties, which rank among the highest in California for asthma‐related health burdens, face disproportionate risks from these events. In this retrospective study, the association between wildfire‐specific PM_2.5_ exposure and asthma‐related hospital encounters was evaluated among children (0–18 years old) in these two counties from January 2017 to February 2020 at a regional children's hospital. Distributed lag quasi‐Poisson regression models were used to assess short‐term impacts up to six days post‐exposure. Over 9,300 hospital visits were modeled and found that wildfire‐specific PM_2.5_ was associated with increasing asthma‐related hospital encounters. Specifically, each 10 μg m^−3^ increase in wildfire‐specific PM_2.5_ was associated with a 4% (95% CI: 0%–8%) increase in pediatric hospital encounter risk on the day of exposure, with additional 3%–4% increases on days 3 and 4 (95% CI: 1%–4% and 1%–6%, respectively). Cumulatively, these exposures corresponded to a 13% (95% CI: 9%–18%) increased risk over the five‐day lag period. Baseline hospital encounter risk varied across census tracts, reflecting underlying community vulnerabilities. Notably, wildfire‐related health risks were amplified in communities with high baseline vulnerability compared to communities with lower vulnerabilities. These findings quantify the risk of wildfire smoke in communities already facing environmental and socioeconomic disadvantages, emphasizing the need for targeted public health interventions in high‐risk areas.

## Plain Language Summary

Wildfire smoke has become a major air pollution problem in the Western U.S., and some communities are more affected than others. This study examined how wildfire smoke affects children's asthma‐related hospital visits in Alameda and Contra Costa Counties in Northern California, using data from a regional children's hospital between January 2017 and February 2020. Results showed that as levels of wildfire‐related air pollution, specifically, fine particles called PM_2.5_, increased, so did the number of asthma‐related hospital visits among children. A 10‐unit increase in wildfire smoke PM_2.5_ was linked to a 4% higher risk of asthma hospital visits on the same day, with elevated risk continuing for several days following the initial smoke event. Over a five‐day period, the total increase in risk was about 13%. The effects of wildfire smoke were more severe in communities already facing environmental and socioeconomic challenges. These findings highlight the need for targeted public health efforts to protect children in the most vulnerable areas during wildfire events.

## Key Points

- Children living in burdened communities experienced increased asthma hospital encounters both day of and days following wildfire smoke exposure
- Baseline asthma risk aligned with CalEnviroScreen indicators reinforcing known patterns of environmental injustice
- Findings emphasize the need for both acute wildfire smoke response and long‐term investment for vulnerable pediatric populations

## 1 Introduction

Over the past decade, wildfire‐specific fine particulate matter (WF PM_2.5_) has increasingly become a significant contributor to the air pollution health burden across the Western United States (US) and beyond. Due to climate change and variability, western environments in North America have become drier and warmer resulting in more frequent and intense wildfire events (Abatzoglou & Williams, 2016; Burke et al., 2020; Mueller et al., 2020; Westerling et al., 2006). In some regions of the US, this increase in wildfire activity has reversed decades of progress in improving air quality, making WF PM_2.5_ the dominant source of seasonal particulate pollution (Burke et al., 2023; Ford et al., 2018).

Mounting evidence shows that WF PM_2.5_ poses substantial health risks, including respiratory, pregnancy‐related, and mental health impacts (Heft‐Neal et al., 2023). In particular, short‐term spikes in WF PM_2.5_ exposure are associated with increases in unplanned healthcare visits and hospitalizations for respiratory‐related conditions (Aguilera, Corringham, Gershunov, & Benmarhnia, 2021; Aguilera et al., 2020; Gould et al., 2024; Henry et al., 2021; Holm et al., 2021; Kondo et al., 2019; Moore et al., 2023; Reid & Maestas, 2019; Zhang et al., 2023). There is also evidence that WF PM_2.5_ may be more harmful than other sources of PM_2.5_ due to its higher oxidative potential and results in a more robust inflammatory and oxidative stress response in the lungs (Aguilera, Corringham, Gershunov, & Benmarhnia, 2021; Borchers Arriagada et al., 2019; Heaney et al., 2022; Horne et al., 2024; Mahsin et al., 2022; Xing et al., 2016).

The health impacts of WF PM_2.5_ exposure can manifest rapidly, with immediate increases in respiratory‐related hospital and emergency department visits often observed during and shortly after exposure periods. The acute nature of these effects highlights the importance of timely public health responses and preventive measures (Borchers Arriagada et al., 2019; Heaney et al., 2022; Horne et al., 2024). However, there is limited evidence of post‐exposure risk on the days following the initial day of smoke presence as few studies have explored the persistence of health risks in the days following the initial exposure.

Certain communities face disproportionately higher risks from wildfire smoke and, more broadly, climate change due to historical and ongoing inequities (Berberian et al., 2022; J. A. Casey et al., 2023; Forastiere et al., 2007; Mohai & Saha, 2015). Under‐resourced and socially marginalized groups often live and work in areas with greater cumulative pollution and may have poorer baseline health (Berberian et al., 2022; J. A. Casey et al., 2024; Chen et al., 2024). These structural factors compound the negative effects of WF PM_2.5_. Children, in particular, are more susceptible to respiratory impacts because of their higher minute ventilation, elevated activity levels, and still‐developing respiratory and immune systems (Heaney et al., 2022; Holm et al., 2021; Horne et al., 2024; Landguth et al., 2024; Li et al., 2023; Mahsin et al., 2022; Moore et al., 2023). A subset of literature exploring health impacts of WF PM_2.5_ shows that it specifically elevates the risk of respiratory issues in children, including asthma exacerbations and other respiratory tract infections (Aguilera, Corringham, Gershunov, Leibel, et al., 2021; Henry et al., 2021; Holm et al., 2021; Landguth et al., 2024; Leibel et al., 2020; Li et al., 2023; Moore et al., 2023; Zhang et al., 2023). Despite these insights, major knowledge gaps persist in fully understanding how WF PM_2.5_ impacts pediatric asthma, particularly in terms of delayed (lagged) effects and differences across socioeconomically diverse communities.

While previous studies have documented the acute effects of wildfire smoke on respiratory outcomes, most have focused on the general population and pooled data across broad geographic areas, limiting insight into localized effects in highly burdened communities. Also, few have examined how these effects persist over multiple days. To address these gaps, this study evaluates the short‐term association between wildfire‐specific PM_2.5_ and pediatric asthma‐related hospital visits, using data from a regional children's hospital serving Alameda and Contra Costa Counties in Northern California, two communities with high asthma burdens and significant structural disadvantage. The timing of these effects was also examined, assessing both day‐of and lagged risks in the days following smoke exposure. By confirming community‐level baseline risk factors with acute exposure metrics, these findings can help inform targeted public health interventions for vulnerable pediatric populations in wildfire smoke impacted regions.

## 2 Methods

**Figure 1:**
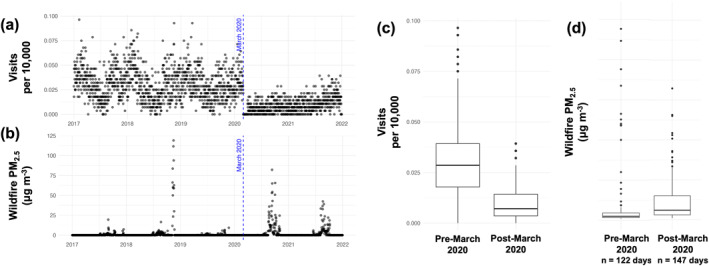
(a, b) time series data of total asthma‐related hospital encounters (per 10,000) per day and daily average wildfire‐specific PM_2.5_ across, respectively, Alameda and Contra Costa counties. (c) Boxplots of total asthma‐related hospital encounters (per 10,000) per day for pre‐March 2020 (left) and post‐March 2020 (right) to show the impact of the COVID‐19 pandemic on asthma hospital encounter rates. (d) Boxplot of wildfire‐specific PM_2.5_ for days when the average wildfire‐specific PM_2.5_ was greater than 1 μg m^−3^ across the two counties pre‐March 2020 (left) and post‐March 2020 (right).

This is a retrospective cohort study assessing the relationship between short‐term exposure to WF PM_2.5_ and acute hospital encounters for asthma at a regional children's hospital. This work covers the period from 1 January 2017–29 February 2020 at University of California San Francisco (UCSF) Benioff Children's Hospital Oakland, using information from all asthma‐related hospital encounters for patients residing in Alameda or Contra Costa Counties (the primary catchment area of the hospital). These counties include neighborhoods and communities that have been designated as disadvantaged due to historical practices and environmental exposure by government protection agency programs. All individuals in this data set were 18 years or younger. The impact of the COVID‐19 pandemic resulted in a dramatic change in the seasonal pattern of asthma‐related hospital encounters and therefore, data during the pandemic (March 2020 onwards) was excluded in this work (see Figure 1). The approach and methods for data extraction were approved by the UCSF IRB #21–33697.

### 2.1 Pediatric Hospital Encounter Records

Outcome data was collected from UCSF Benioff Children's Hospital Oakland, a regional Level 1 trauma center and referral destination for children in Northern California to the Southern Oregon border. Extracted data from the electronic health record (EHR) included address data, ICD codes, encounter type, and encounter date and timestamp. Addresses were geocoded and filtered to exclude records associated with P.O. box addresses rather than physical addresses. The data set was then narrowed to hospital encounters specifically for asthma, identified by the ICD code J45.X, within the study period restricted from 1 January 2017 to 29 February 2020. This process resulted in a final data set comprising 9,338 encounters of asthma‐related hospital encounters.

### 2.2 Wildfire‐Specific PM_2.5_ Exposure Data

The daily WF PM_2.5_ data set used for exposure assignment was resolved at the census tract level (Aguilera et al., 2023; J. Casey et al., 2024; J. A. Casey et al., 2024). Daily all‐source PM_2.5_ was estimated using a machine learning ensemble model, which integrated PM_2.5_ data from monitoring stations with relevant predictors such as ground‐level, observational data, satellite‐derived data, meteorology, and land‐use information (Aguilera et al., 2023). Machine learning models were evaluated by reserving five monitoring sites across California as an independent hold‐out test set, while using the remaining monitoring data to training and validation base learner models, specifically 80% for training and 20% for 10‐fold cross validation. The ensemble model achieved an *R* ^2^ of approximately 0.78 and a RMSE of 3.51 μg m^−3^ for predicting all‐source PM_2.5_ (Aguilera et al., 2023). They then identified smoke‐impacted tract‐days by intersecting census tracts with NOAA Hazard Mapping System (HMS) smoke‐plume polygons. For those smoke‐impacted tract‐days the authors treated the non‐smoke (background) PM_2.5_ as a missing counterfactual and applied a chained random‐forest multiple‐imputation procedure (i.e., iterative conditional models) using the same predictor set to generate multiple plausible non‐smoke PM_2.5_ values per missing observation. The multiple imputations were averaged to produce a single counterfactual baseline PM_2.5_ for each smoke‐impacted tract‐day. The out‐of‐bag error from the chained random‐forest multiple imputation approach was ∼0.78% (Aguilera et al., 2023). WF PM_2.5_ was determined as the difference between total daily PM_2.5_, from the ensemble model, and the imputed values regarding counterfactual non‐wildfire smoke‐specific PM_2.5_. The daily average distributions of total PM_2.5_, non‐wildfire smoke‐specific PM_2.5_, and wildfire smoke‐specific PM_2.5_ can be found in Figure S1 of Supporting Information S1 and shows a long tail of higher concentrations for wildfire smoke‐specific PM_2.5_ compared to the other two distributions. For this study, each asthma hospital encounter outcome was assigned a mean daily WF PM_2.5_ concentration for the day of the outcome as well as for the 6 days preceding the outcome using the census tract of residence on the date of hospital encounter.

### 2.3 Weather and Other Covariates

The daily minimum temperature (°C) at census tract resolution was used as a covariate in the models in this study. Minimum temperature is commonly used to account for the impact of extreme heat on respiratory outcomes due to air pollution. Temperature data was sourced from University of Idaho Gridded Surface Meteorological Data set (Abatzoglou, 2013). Variation due to season was addressed with a spline for time as described below.

### 2.4 Geospatial Data on Baseline Characteristics

Descriptive statistics were used to compare study characteristics and baseline risk values with indicators from CalEnviroScreen 4.0 (CES) to assess consistency with known environmental and socioeconomic risk factors in the region. CES was developed by the California Environmental Protection Agency and is used to identify highly burdened populations (those with both social disadvantages and elevated pollutant exposures) (California Office of Environmental Health Hazard Assessment (OEHHA), 2021). It has a cumulative indicator of elevated burden due to environmental exposures for populations across California. Overall, poverty, and asthma CES indicators at the census tract level were extracted as these all relate to the exposure‐outcome relationship explored in this. The overall CES score is calculated by multiplying the pollution burden score, which averages exposures and environmental effects indicators, with the population characteristics score, which averages sensitive population and socioeconomic factors. The asthma indicator represents the rate of asthma‐related emergency department visits per 10,000 residents, averaged over the years 2015–2017. The poverty indicator correlates to the percent of the population with incomes less than two times the federal poverty level.

Tract‐specific baseline risk for asthma‐related hospital encounter was estimated through evaluating the random effect at the census tract level from the model. This random intercept allowed us to capture and quantify the inherent differences in baseline risk across various census tracts, accounting for unobserved, time‐fixed characteristics unique to each tract. This method enabled the identification of spatial patterns of baseline risk that are independent of the exposure to wildfire smoke‐specific PM_2.5_ and other covariates. The tract‐specific baseline risk extracted from the random intercepts was subsequently normalized to the mean across all census tracts to facilitate a comparative analysis. This normalization step provided a clearer understanding of how each tract's baseline risk deviates from the average risk across the study area. Evaluation on the level of agreement between CES indicators and this model's baseline risk metrics were done using a spearman correlation statistic.

### 2.5 Modeling Asthma‐Related Hospital Encounters as a Function of Wildfire Smoke

This analysis applies a dynamic modeling framework to inform on the short‐term (<1 week) association between the exposure, WF PM_2.5_, and the health outcome, asthma‐related pediatric hospital encounters. The association between WF PM_2.5_ concentration and daily counts of asthma‐related hospital visits were assessed using a distributed lag model (DLM), which models the effects of the exposure in a way that robustly controls for the exposures that occur at other lags (Gasparrini et al., 2010). Such a DLM can accurately capture the cumulative and lagged relationships between wildfire smoke exposure and asthma‐related hospital encounters.

In this study, the exposure was assessed as a continuous measurement and the outcome as a count variable (allowing for potential overdispersion). The DLM used a 6‐day lag with a quasi‐Poisson regression assuming a log‐linear relationship between WF PM_2.5_ and daily asthma hospital counts, see Equation 1 (Kelsall et al., 1999). Quasi‐Poisson regression was used because the outcomes were recorded as counts and over dispersed, which requires a modification on the traditional Poisson distribution (Table S1 in Supporting Information S1). A basic spline function was used to handle temporal variation and seasonal confounding (bs(t), degrees of freedom = 7 per year). Natural and basic splines and varying degrees for both spline types were tested before determining the one based on the lower Akaike Information Criteria (AIC) value and commonly used practices for seasonal modeling (Table S2 in Supporting Information S1). Temperature (Temp) was included in the model based on an optimized cross‐basis function to consider non‐linear relationships and lagged effects. A random effect for the intercept at the census tract level (β_c_) was included in the model to account for within census tract time‐fixed confounders. A census‐tract specific population offset (*p*_c_) was also used, however, population counts did not change over time. To assess the sensitivity of the model, several alternative specifications were tested. First, a version of the model was estimated without including a random effect for census tract. Second, the primary model was stratified by overall CES quartiles, such that separate models were run for each quartile group based on the patients' census tract of residence. Additionally, the original model was run with data post‐March 2020 to account for potential temporal variations due to the pandemic behavior response and with data specifically from either Alameda or Contra Costa County to test for spatial differences in risks between counties.

(1) $$\mathrm{Log}E\left(Y_{t,c}\right)=\sum_{l=0}^{6}\beta_{WF,l}{\text{WFPM}}_{t-l}+\sum_{l=0}^{6}{\beta_{\text{Temp},l}\text{Temp}}_{t-l}+\text{bs}\left(t\right)+p_{c}\;{+\beta }_{c}+\beta_{0}$$

Equation 1 represents a distributed 6‐day lag model with random effect for census tract and consists of a crossbasis for wildfire smoke‐specific PM_2.5_ (WFPM), a crossbasis for temperature (Temp), basic spline for time (bs(t), 7 degrees of freedom per year), census tract‐specific population offset (*p*_c_), census tract‐specific random intercept (β_c_), and overall intercept (β_0_).

## 3 Results

### 3.1 Study Wildfire Smoke and Population Characteristics

The study period (January 2017–February 2020) included three distinct wildfire periods with daily wildfire‐specific PM_2.5_ concentrations ranging from ∼0 to 125 μg m^−3^. For days with spatially averaged wildfire‐specific PM_2.5_ greater than 1 μg m^−3^ across the two counties, the median WF PM_2.5_ concentration was ∼3 μg m^−3^, heavily skewed by typically lower concentrations as only a few days experiences high pollution levels (Figure 1).

This study population represents a diverse population with higher rates of non‐white pediatric patients including 36% Non‐Hispanic Black or African American and 38% Hispanic or Latino children (Table 1). Approximate 61% of the study population were boys and the median age was 7 years (Q1–Q3: 4–12). The study population resided in 484 census tracts within the two counties, Alameda and Contra Costa. In these counties, approximately 82% of the study population live in tracts with >20% of the population two times below the federal poverty level. Further, 74% of the study population resides in census tracts that were in third and fourth quartile for overall CES score, reflecting higher levels of environmental and socioeconomic burden than compared to the state average.

**Table 1:** Study Population Characteristics of Hospital Encounters for Asthma From January 2017–February 2020

Study characteristic	n (%)
*Race/Ethnicity* ^a^	
Asian	479 (6%)
Black or African American	3,348 (36%)
Native American/Alaska Native	7 (<1%)
Native Hawaiian	1 (<1%)
Other	1,124 (12%)
Pacific Islander	80 (<1%)
Non‐Hispanic White	517 (6%)
Hispanic/Latino^b^	3,503 (38%)
*Sex*	
Female	3,625 (39%)
Male	5,713 (61%)
*CES Overall Score Quartile*	
Q1 (CES Score ≤14.79)	840 (9%)
Q2 (14.79 < CES Score ≤25.55)	1,632 (17%)
Q3 (25.55 < CES Score ≤40.06)	3,735 (40%)
Q4 (CES Score > 40.06)	3,131 (34%)
*Total Asthma‐related Visits*	9,338

^a^ <1% Asian, Native American/Alaska Native, Pacific Islander report Hispanic or Latino ethnicity.

^b^ Includes those that indicated “Other” or “White” as race, with Hispanic or Latino ethnicity.

### 3.2 Baseline Risk Mapping

**Figure 2:**
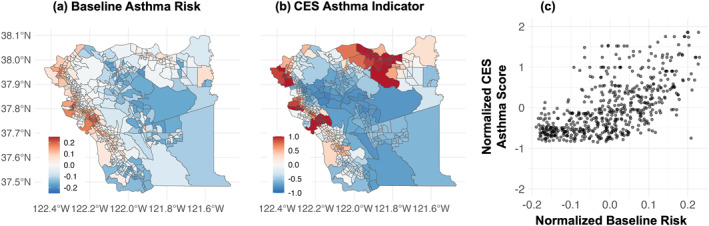
(a, b) Maps of Contra Costa and Alameda Counties colored by normalized baseline risk from this study's model and CalEnviroScreen (CES) asthma indicator, respectively. (c) Scatter plot of normalized baseline risk versus normalized CES asthma indicator score.

Mapping of the normalized tract‐specific baseline risk values for asthma hospital encounters highlight that areas with higher risk reside along the west side of Alameda and Contra Costa Counties (Figure 2a). When compared to the normalized CES asthma indicator, both denote elevated risk areas along the west side of the counties, specifically in the cities of Richmond, Berkeley, West Oakland, and East Oakland (North to South) (Figures 2a and 2b). The Spearman correlation coefficients between the baseline risk and CES asthma indicator values is 0.64 (p‐value < 0.05) implying a significant monotonic relationship. However, the CES asthma indicator is elevated in the northern region of Contra Costa County, whereas the baseline risk from this work does not reflect that increased risk.

The baseline risk similarly aligns with the CES overall and poverty indicators, with Spearman correlation coefficients of 0.61 and 0.68 (p‐value < 0.05), respectively (Figure S2 in Supporting Information S1). For tracts with high CES asthma scores (normalized > 1), baseline asthma hospital encounter risk increases with the CES asthma indicator, indicating a roughly linear relationship. In contrast, among tracts with average or low scores (normalized < 1), CES asthma values remain relatively stable and show little association with baseline risk (Figure 2c).

### 3.3 Exposure‐Response Relationships

**Figure 3:**
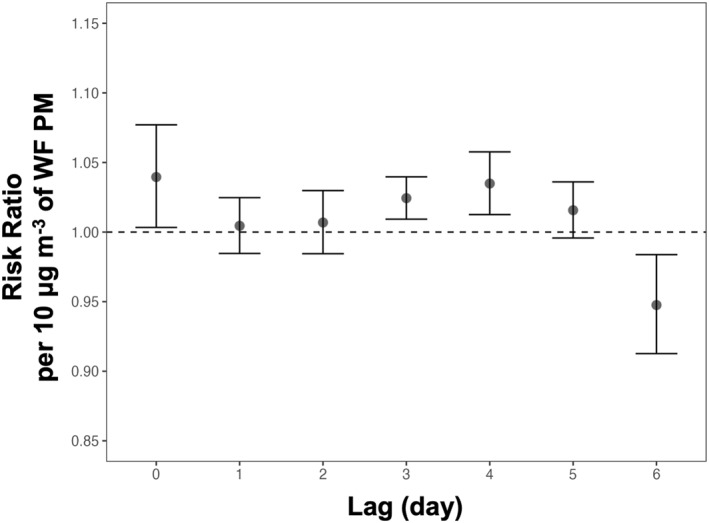
Risk ratio estimates per 10 μg m^−3^ of WF PM_2.5_ per lag day. These estimates were calculated using a distributed lag model with random effect for census tract. Error bars represent a 95% confidence interval.

In the fully adjusted model, for each 10 μg m^−3^ increase in daily average WF PM_2.5_ exposure saw a 4%, 2%, and 4% increase in the risk ratio (RR) of asthma‐related hospital visits for children on each of days 0, 3, and 4 following wildfire‐specific PM_2.5_ exposure (95% CI: 0%–8%, 1%–4%, 1%–6%, respectively, Figure 3 and Table S3 in Supporting Information S1). The cumulative RR peaked after 5 days following a wildfire smoke exposure, days 0–5, at 1.13 (95% CI: 1.09, 1.18). In a sensitivity analysis, where a random effect for census tract is not included, demonstrated a similar pattern with lag days 0, 3, and 4 associated with elevated risk of asthma‐related hospital encounter that was higher in magnitude than seen with the DLM with random effect (Figure S3 in Supporting Information S1).

Additional sensitivity analyses were completed to show the robustness of these results. In a model run with data from post‐March 2020, a similar elevated risk pattern on the day of exposure as well as the days 4 and 5 after exposure was seen (Figure S5 in Supporting Information S1). However, the magnitudes of the risk are overall lower and the corresponding confidence intervals are larger most likely due to the lower rates of visits during this pandemic period (Figure 1). Similarly, when running this model on the individual counties (Alameda and Contra Costa, Figures S6 and S7 in Supporting Information S1, respectively), the magnitude of risk tends to be lower, which may reflect the lower outcome count due to disaggregating, yet, the general pattern of elevated risk remains. For example, for Alameda, there is a significant elevated risk on Day 3 of exposure (RR = 1.02; 95% CI, 1.00–1.04), nearly the same as the full model results.

As an exploratory analysis, the potential heterogeneity in wildfire smoke‐associated risk by CES quartile was examined, which captures baseline community vulnerability (Figure S4 in Supporting Information S1). While limited sample sizes restrict the precision of some estimates, particularly in the lower quartiles (Table 1), this stratified analysis suggests possible differences in the magnitude and timing of risk across communities with varying levels of social and environmental characteristics. These findings point toward potential effect modification by baseline community characteristics, though further investigation with larger sample sizes is warranted to confirm and clarify these patterns.

## 4 Discussion

### 4.1 Study Findings

The distributed lag model revealed that pediatric asthma risks increased significantly on both the day of wildfire smoke exposure (lag 0) and again on subsequent days, with the highest risk observed on day 4 post‐exposure. This delayed peak may reflect underlying inflammatory processes or delayed healthcare‐seeking behavior. Interestingly, a significant decrease in risk on day 6 was also observed, which may indicate behavioral adaptations, such as staying indoors or mask use, or statistical variability. Further investigation is needed to clarify the mechanisms behind this observed dip in risk.

These results align with other studies showing elevated respiratory risks from wildfire smoke, although this study's effect estimates differ in magnitude. For instance, Aguilera et al. (2021) reported a 30% increase in pediatric emergency department visits per 10 μg m^−3^ of WF PM_2.5_, substantially higher than these findings (13% cumulative risk over days 0–5) (Aguilera, Corringham, Gershunov, Leibel, et al., 2021). Differences in population characteristics, measurement methods, and regional healthcare‐seeking behaviors may contribute to these discrepancies. In a systematic review of six studies investigating the association between fire smoke fine particulate matter and asthma‐related outcomes, they found that exposure to landscape fire smoke PM_2.5_ was associated with 1.03 (95% CI: 0.98, 1.08) times higher relative risk for asthma‐related hospital encounter, which was slightly lower than our finding (Borchers Arriagada et al., 2019). The same study found that risk estimates were higher for same day of smoke exposure (lag 0) compared to 1, 2, and 3 days after exposure, but were positive in all situations. In our work, only significant positive associations were found for lag days 0 and 3. The consistent detection of delayed health impacts across multiple studies underscores the importance of monitoring both immediate and lagged effects of wildfire smoke on pediatric populations.

This analysis confirmed that historically disadvantaged neighborhoods, including those in Richmond, West Oakland, and East Oakland, face additional risk of asthma‐related hospital encounter due to WF PM_2.5_. These findings closely mirrored external risk maps, reinforcing that systemic inequalities and historical underinvestment contribute to heightened vulnerability. Indeed, these three communities have been recognized by the California Air Resources Board under Assembly Bill 617 as overburdened communities, highlighting the need for targeted emission reduction plans and additional resources. However, the northern section of Contra Costa County did not have alignment between the baseline risk in this study and the external risk maps, likely reflecting limited patient representation from that region in this single‐hospital data set. This underscores how referral patterns and healthcare access can influence observed spatial patterns.

Further, these results emphasize the WF PM_2.5_ risk i.e. in additional to baseline risk in communities already facing socioeconomic challenges and historical environmental injustices. Housing segregation, redlining, and limited infrastructure investment have resulted in poorer housing quality and less access to protective resources (e.g., air filtration, masks), intensifying exposure to wildfire‐specific PM_2.5_ (Burke et al., 2022; Jacobs, 2011; Liang et al., 2021; Sparks et al., 2024; Swope & Hernández, 2019). In turn, these factors likely exacerbate asthma outcomes among children in these areas. Further, these spatial results are consistent with the “double jeopardy” riskscape, whereby place‐based psychosocial stressors interact with pollutant exposures to increase population susceptibility (Morello‐Frosch & Shenassa, 2006). Meaning, wildfire‐specific PM_2.5_ can produce larger health effects in historically disadvantaged neighborhoods. These observations underscore the need for holistic, community‐centered responses that combine targeted exposure‐reduction measures (e.g., air purifiers, outreach, stricter emissions controls) with policies addressing upstream social determinants (e.g., housing, healthcare access, chronic stress) to mitigate these risks and advancing health equity.

### 4.2 Study Limitations and Future Directions

Despite the strengths of this study, several limitations warrant consideration. First, the reliance on data from a single hospital may constrain the generalizability of these findings to other regions or healthcare settings. The patient population included here may not fully represent all individuals affected by wildfire smoke in the broader study area. Although this model adjusted for some confounding factors, unmeasured variables, such as individual behaviors during smoke events (e.g., use of air filtration systems, mask‐wearing, or choosing to stay indoors), may have influenced the observed associations. Additionally, as the exposure data was derived by an ensemble model with some errors, the results of the health analysis propagate these errors into the risk estimates, therefore there is potential that these estimates are not fully certain even within their confidence intervals based on the original exposure uncertainties.

Future research should address these limitations by expanding the geographic scope and incorporating multiple healthcare systems to validate and extend this study's findings. Larger, longitudinal studies that integrate personal‐level exposure metrics, community characteristics, and healthcare utilization data are needed to capture the complexities of WF PM_2.5_‐related asthma exacerbations among children. Studies focusing on specific racial or marginalized populations are particularly important for clarifying how these groups respond to wildfire smoke exposure compared to the general population. Integrating qualitative methods could also provide valuable insights into behavioral adaptations and community‐level responses, informing more nuanced public health strategies. Given the projected increase in wildfire frequency and severity under climate change, understanding and mitigating the health impacts of wildfire smoke, especially in vulnerable pediatric populations, will become ever more urgent.

## 5 Conclusion

In summary, this study demonstrates that children from historically disadvantaged communities with elevated baseline risks not only face chronic vulnerabilities but also experience significantly amplified adverse health outcomes when exposed to wildfire‐specific PM_2.5_. These results consider long‐standing environmental disparities and acute exposures, underscoring the urgent need for integrated public health strategies that protect pediatric populations. Efforts to reduce chronic environmental burdens, alongside acute smoke event management, are essential to safeguarding children's respiratory health. As climate change intensifies wildfire activity, prioritizing both chronic risk reduction and acute exposure mitigation will be critical to improving health outcomes and advancing environmental justice.

## Conflict of Interest

The authors declare no conflicts of interest relevant to this study.

## Supporting information

Supporting Information S1

## Data Availability

The census tract‐level wildfire‐specific PM_2.5_ data used for exposure in this study are publicly available (J. Casey et al., 2024). Temperature data sourced from University of Idaho Gridded Surface Meteorological Data set (gridMET) and made publicly available (https://www.climatologylab.org/gridmet.html) (Abatzoglou, 2013). The census tract‐level community risk metrics are available from CalEnviroScreen version 4.0 (https://oehha.ca.gov/calenviroscreen/report/calenviroscreen‐40) (California Office of Environmental Health Hazard Assessment (OEHHA), 2021). The pediatric asthma‐related hospital encounter level data from the University of California San Francisco (UCSF) Benioff Children's Hospital Oakland are not publicly available to protect patient privacy and comply with institutional data use agreements. Researchers who meet the criteria for access to confidential data (e.g., completion of human subjects training and data use agreements) may request access through the UCSF Institutional Review Board. Requests for data access should be directed to Neeta Thakur (neeta.thakur@ucsf.edu). Models, figures and maps were created in R version 4.3.2 (31‐10‐2023) using the packages “ggplot2” (version 3.5.1), “sf” (version 1.0–15), and “dlnm” (Gasparrini, 2011), along with base R graphics. Software and packages are freely available from the Comprehensive R Archive Network (CRAN; https://CRAN.R‐project.org).
