# Supplementary material for: Wildfire‐Specific PM2.5 Asthma Risk Across a Disproportionately Burdened Pediatric Population in Northern California
Source: Geohealth. 2026 Feb 11;10(2):e2025GH001530. doi: 10.1029/2025GH001530 (PMC12892088; doi:10.1029/2025GH001530)
Supplement: Supplementary file 1 — Supporting Information S1 [file GH2-10-e2025GH001530-s001.pdf]

[GeoHealth]

Supporting Information for

[Wildfire-Specific $PM_{2.5}$ asthma risk across a disproportionately burdened pediatric population in Northern California]

[Rebecca Sugrue[1]; Stephanie Holm[,2]; Andrew Nguyen[1]; Morgan Ye[1]; Rosana Aguilera[3]; Dayna Long[4]; Tarik Benmarhnia[3]; Rosemarie De La Rosa[5]; Neeta Thakur[1]]

[[1]Department of Medicine, University of California San Francisco, San Francisco, CA
[2]Stephanie Holm Consulting, Vancouver, BC, Canada
[3]Scripps Institution of Oceanography Department, University of California San Diego, San Diego, CA
[4]Department of Pediatrics, University of California San Francisco, San Francisco, CA
[5]School of Public Health, University of California Berkeley, Berkeley, CA]

Contents of this file

Figures S1 to S7

Tables S1 to S3

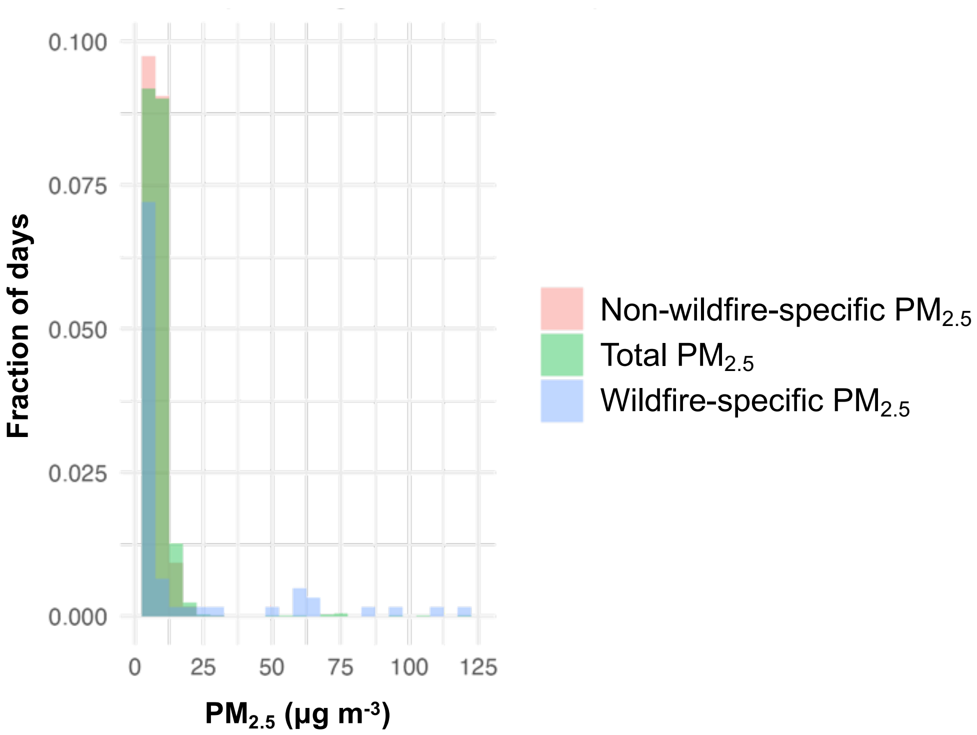

Figure S1. Histogram of $PM_{2.5}$ concentration distributions for total $PM_{2.5}$, non-wildfire-specific $PM_{2.5}$, and wildfire-specific $PM_{2.5}$. Note, these distributions exclude concentrations equal to 0 μg m^{-3}.

Table S1. Statistics of asthma hospitalization visits per census tract per day.

**Minimum**	0
**1st Quartile**	0
**Median**	0
**3rd Quartile**	0
**Maximum**	4
**Mean**	0.014

Table S2. QAIC values for time splines. This model optimized for the local minima of the basic spline (degree = 3) df = 8 for time to account for seasonal confounding.

df	Basic spline (degree = 3)	Basic spline (degree = 2)	Natural Spline
2	91278.98	91280.7	91281.34
3	91278.98	91279.07	91281.05
4	91283.97	91279.05	91282.21
5	91305.85	91300.58	91281.13
6	91416.25	91391.99	91321.18
7	91401.61	91429.13	91474.35
8	91232.54	91230.02	91315.11
9	91411.79	91375.98	91353.98
10	91325.45	91315.23	91346.85
11	91371.9	91321.36	91271.6
12	91344.47	91326.43	91342.01

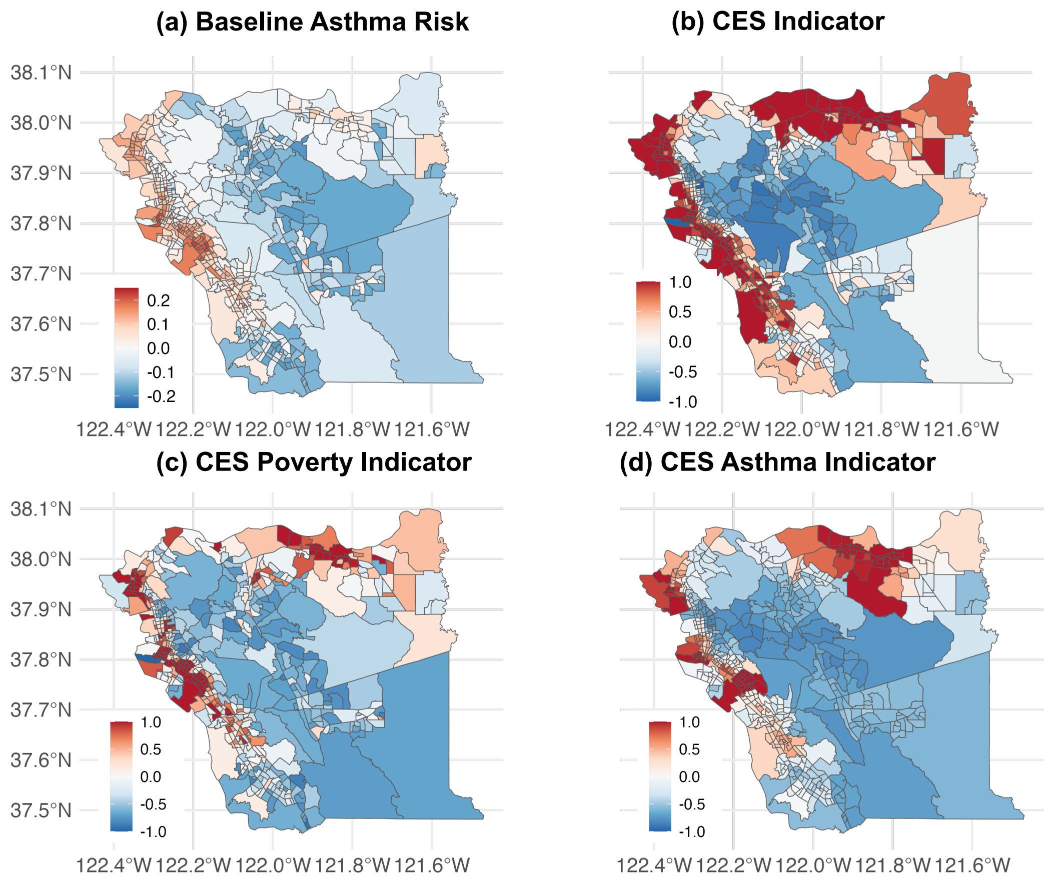

Figure S2. Maps of Contra Costa and Alameda Counties colored by normalized (a) baseline asthma hospitalization risk from our study's model and CalEnviroScreen 4.0 (CES) (b) overall, (c) poverty, and (d) asthma indicators.

Table S3. Risk ratio estimates per 10 µg m^{-3} of WF $PM_{2.5}$ per lag day (Day). These estimates were calculated using a distributed lag model with random effect for census tract. Days 0, 3, and 4 (bolded) were found to have significant increase in risk based on the confidence interval, also listed in the table.

Day	RR per 10 µg-m^{-3} WF PM2.5	Confidence interval (95%)
**0**	**1.04**	**(1.00, 1.08)**
1	1.00	(0.98, 1.02)
2	1.01	(0.98, 1.03)
**3**	**1.02**	**(1.01, 1.04)**
**4**	**1.04**	**(1.01, 1.06)**
5	1.02	(0.99, 1.04)
6	0.95	(0.91, 0.98)

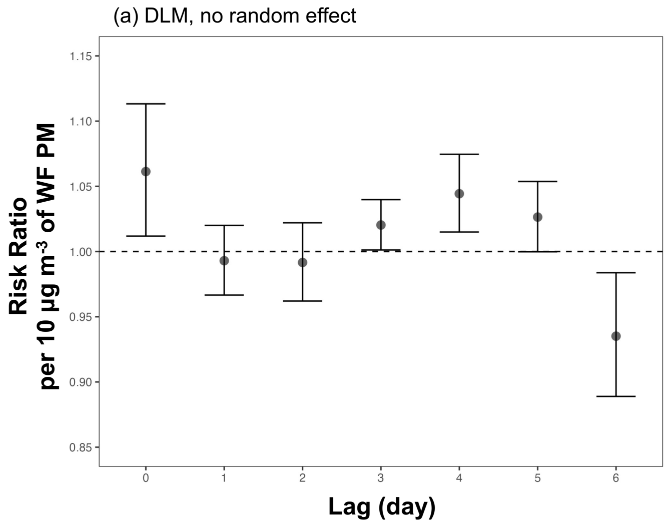

Day	RR per 10 µg-m^{-3} WF $PM_{2.5}$	Confidence interval (95%)
**0**	**1.06**	**(1.01, 1.11)**
1	0.99	(0.97, 1.02)
2	0.99	(0.96, 1.02)
**3**	**1.02**	**(1.00, 1.04)**
**4**	**1.04**	**(1.01, 1.07)**
5	1.02	(0.99, 1.05)
6	0.93	(0.89, 0.98)

Figure S3. (a) Risk ratio (RR) estimates per 10 µg m^{-3} of WF $PM_{2.5}$ per lag day. These estimates were calculated using a distributed lag model (no random effect). Error bars represent a 95% confidence interval and (b) table of corresponding RRs per lag day with Days 0, 3, and 4 (bolded) to have significant increase in risk based on the confidence interval.

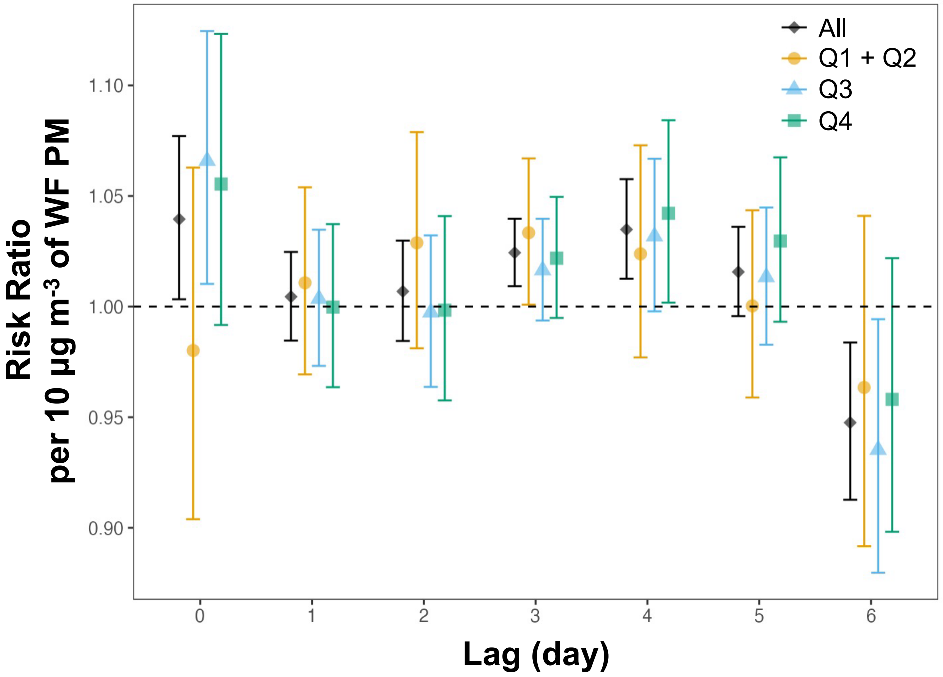

Figure S4. Risk ratio estimates per 10 µg m^{-3} increase in wildfire-related $PM_{2.5}$ by lag day, stratified by CES overall indicator quartile as well as for the original model with all the data. Estimates were derived from a distributed lag model with a random effect for census tract. Due to small sample sizes in Quartile 1 (Q1), Q1 and Q2 were combined into a single group. Error bars represent 95% confidence intervals.

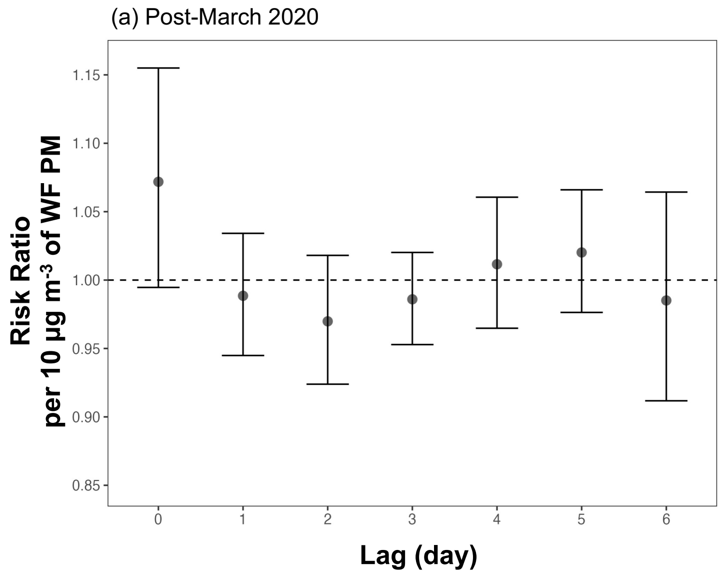

Day	RR per 10 μg-m^{-3} WF $PM_{2.5}$	Confidence interval (95%)
0	1.06	(0.99, 1.16)
1	0.99	(0.94, 1.03)
2	0.97	(0.92, 1.02)
3	0.99	(0.95, 1.02)
4	1.01	(0.96, 1.06)
5	1.02	(0.98, 1.06)
6	0.99	(0.91, 1.06)

Figure S5. (a) Risk ratio (RR) estimates per 10 μg m^{-3} of WF $PM_{2.5}$ per lag day. These estimates were calculated using a distributed lag model with random effect on data from post-March 2020. Error bars represent a 95% confidence interval and (b) table of corresponding RRs per lag day.

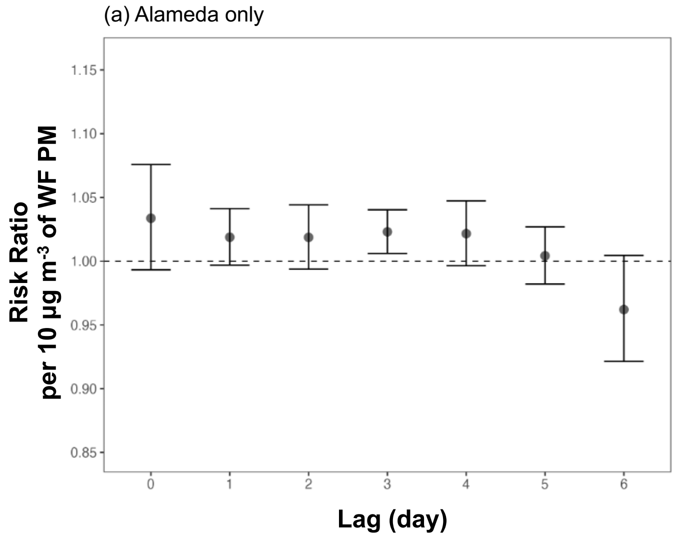

Day	RR per 10 μg-m^{-3} WF $PM_{2.5}$	Confidence interval (95%)
0	1.03	(0.99, 1.08)
1	1.02	(0.99, 1.04)
2	1.02	(0.99, 1.04)
**3**	**1.02**	**(1.00, 1.04)**
4	1.02	(0.99, 1.05)
5	1.00	(0.98, 1.03)
6	0.96	(0.92, 1.00)

Figure S6. (a) Risk ratio (RR) estimates per 10 μg m^{-3} of WF $PM_{2.5}$ per lag day. These estimates were calculated using a distributed lag model with random effect on data from only Alameda County. Error bars represent a 95% confidence interval and (b) table of corresponding RRs per lag day with Day 3 (bolded) having a significant increase in risk based on the confidence interval.

(a) Contra Costa only

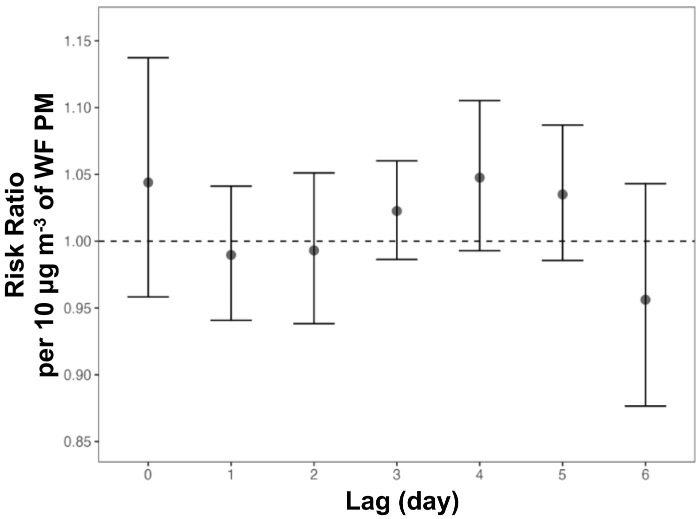

Day	RR per 10 µg-m^{-3} WF $PM_{2.5}$	Confidence interval (95%)
0	1.03	(0.96, 1.14)
1	0.99	(0.94, 1.14)
2	0.99	(0.94, 1.05)
3	1.02	(0.98, 1.06)
4	1.05	(0.99, 1.10)
5	1.03	(0.98, 1.08)
6	0.96	(0.87, 1.04)

Figure S7. (a) Risk ratio (RR) estimates per 10 µg m^{-3} of WF $PM_{2.5}$ per lag day. These estimates were calculated using a distributed lag model with random effect on data from only Contra Costa County. Error bars represent a 95% confidence interval and (b) table of corresponding RRs per lag day.
